# RNF20 Is Critical for Snail-Mediated E-Cadherin Repression in Human Breast Cancer

**DOI:** 10.3389/fonc.2020.613470

**Published:** 2020-12-08

**Authors:** Danping Wang, Yifan Wang, Xuebiao Wu, Xiangxing Kong, Jun Li, Chenfang Dong

**Affiliations:** ^1^ Department of Pathology and Pathophysiology, and Department of Surgical Oncology of the Second Affiliated Hospital, Zhejiang University School of Medicine, Hangzhou, China; ^2^ Key Laboratory of Disease Proteomics of Zhejiang Province, Zhejiang University School of Medicine, Hangzhou, China; ^3^ Cancer Institute of Integrative Medicine, Zhejiang Academy of Traditional Chinese Medicine, Tongde Hospital of Zhejiang Province, Hangzhou, China; ^4^ Department of Pathophysiology, Zunyi Medical University, Zunyi, China

**Keywords:** epithelial-mesenchymal transition, snail, RNF20, ubiquitination, G9a

## Abstract

**Background:**

E-cadherin, a hallmark of epithelial-mesenchymal transition (EMT), is often repressed due to Snail-mediated epigenetic modification; however, the exact mechanism remains unclear. There is an urgent need to understand the determinants of tumor aggressiveness and identify potential therapeutic targets in breast cancer.

**Experimental design:**

We studied the association of RNF20 with Snail and G9a by co-immunoprecipitation. We employed quantitative real-time PCR, ChIP, transwell assay, colony formation assay, and mammosphere assay to dissect the molecular events associated with the repression of E-cadherin in human breast cancer. We used a proteogenomic dataset that contains 105 breast tumor samples to determine the clinical relevance of RNF20 by Kaplan-Meier analyses.

**Results:**

In this study, we identified that Snail interacted with RNF20, an E3 ubiquitin-protein ligase responsible for monoubiquitination of H2BK120, and G9a, a methyltransferase for H3K9me2. RNF20 expression led to the inhibition of E-cadherin expression in the human breast cancer cells. Mechanically, we showed that RNF20 and H3K9m2 were enriched on the promoter of E-cadherin and knockdown of Snail reduced the enrichment of RNF20, showing a Snail-dependent manner. RNF20 expression enhanced breast cancer cell migration, invasion, tumorsphere and colony formation. Clinically, patients with high RNF20 expression had shorter overall survival.

**Conclusion:**

RNF20 expression contributes to EMT induction and breast cancer progression through Snail-mediated epigenetic suppression of E-cadherin expression, suggesting the importance of RNF20 in breast cancer.

## Introduction

Breast cancer metastasis is a major problem that causes high mortality in cancer patients (1). About 90% of cancer deaths result from the local invasion and distant metastasis of tumor cells (2–5). Therefore, a better understanding of the molecular events that contribute to tumor invasiveness is crucial to the development of therapeutic strategies.

EMT is a complex process in which epithelial cells lose contact with their neighbors, and possess the phenotype of migratory mesenchymal cells during in embryonic development, wound healing, tissue remodeling and tumor metastasis (6, 7). EMT promotes tumor progression by endowing tumor cells with the properties of CSCs. E-cadherin loss is a hallmark of EMT (5). Snail, a crucial regulators of E-cadherin, can downregulate the expression of E-cadherin by mediating histone modification (6). Histone modifications are crucial in regulating basic processes including transcription, cell differentiation, cell cycle progression, and DNA repair (8–11). RNF20 is known to be required to regulate chromosome structure by monoubiquitinating histone H2B at lysine 123 (H2BK123) in budding yeast and at lysine 120 (H2BK120) in humans (12, 13). H2BK120ub is a key histone modification that plays critical roles in gene transcriptional regulation and higher order chromatin organization in many species (8, 14–20). Ubiquitination of histone H2B (H2Bub) has been reported to be associated with highly expressed active genes in human cells (15, 21). However, recent report has shown that RNF20, presumably *via* H2Bub, selectively represses expression of some genes by inhibiting TFIIS binding to chromatin (22).

In this study, we have determined that RNF20 interacts with Snail and G9a and is recruited to the E-cadherin promoter to represses its expression, leading to EMT induction.

## Materials and Methods

### Cell Culture

All cancer cell lines were grown in DMEM/F12 supplemented with 10% FBS, except breast cancer cell lines BT549 and BT474, which were grown in RPMI-1640 plus 10% FBS. For establishing stable transfectants with overexpression of RNF20, MDA-MB231-Luc-D3H1 (with stable expression of luciferase, from Xenogene Corp.) and BT549 cells were transfected with G9a-PLVX; stable clones were selected with puromycin (300 ng/ml) for 4 weeks.

### Plasmids, shRNA, and Antibodies

RNF20 shRNA expression plasmids were purchased from MISSION shRNA at Sigma-Aldrich. The catalog numbers of shRNAs were TRCN0000437632 and TRCN0000033878. The plvx-neo-RNF20 contained silent mutation which was resistant to shRNF20-1. The sequence of the mutation was 5’-CAAAGGTTAAATAGG CATCTCG-3’. Expression plasmids for RNF20, Snail were provided by Arthur Danping Wang. Human RNF20 and Snail was amplified from a MDA-MB231 cDNA library and subcloned into vector plvx(puro). Antibodies against HA, Flag, and actin were purchased from Sigma-Aldrich. Antibodies for E-cadherin and β-actin were from BD Transduction Laboratories. RNF20 antibodies and Snail antibodies were from Abcam and Cell Signaling Technology respectively.

### Quantitative Real-Time PCR

Total RNA was isolated using the TRIzol Reagent (ThermoFisher scientific) according to manufacturer’s instructions. Specific quantitative real-time PCR experiments were performed using SYBR Green Power Master Mix following manufacturer’s protocol (Applied Agbio, Foster City, Changsha, CHN). The qPCR primers of E-cadherin were 5’-TGCCAACTGGCTGGAGATTA-3’ and 5’-AGTGTCCCTGTTCCAGTAGC-3’. The qPCR primers of RNF20 were 5’-AAAGCATCGCACCATGTCTC-3’ and 5’-ATCCCACTGCAGGTCATCAA-3’.

### Migration and Invasion Assays

Migration and invasion assays were performed in Boyden chambers as described previously (23). The cancer cells were stained and counted with a microscope. All experiments were performed at least twice in triplicate. Statistical analysis was performed using Student’s t test; a p-value of <0.05 was considered significant.

### Co-Immunoprecipitation

Co-IP assays were performed as described previously (24). Briefly, cells were lysed in lysis buffer that contained a cocktail of protease inhibitors. After being adjusted to equal protein concentration, samples were mixed with 4× SDS–PAGE sample buffer and boiled for 5 min. The pull-down complexes were examined by Western blotting.

### Chromatin Immunoprecipitation

ChIP assays were performed as described previously (23). The primers for the E-cadherin promoter were 5’-ACTCCAGGCTAGAGGGTCACC-3’ and 5’-CCGCAAGCTCACAGGTGCTTTGCAGTTCC-3’ (25). The cells were prepared to perform ChIP assay with the Imprint ChIP kit (Sigma-Aldrich) according to the manufacturer’s instructions as described recently.

### Colony Formation Assay

Colony formation assay was performed using double-layer soft agar in 24-well plates with a top layer of 0.35% agar and a bottom layer of 0.7% agar. Cells were seeded into 24-wellplates in the desired medium and cultured at 37°C for 20 days, and the colonies were stained and counted.

### Mammosphere Assay

Mammosphere assays were performed following the protocol previously described by plating single-cell suspensions into ultralow-attachment 6-well plates (Corning) in mammosphere culturing conditions and counting after 7-14d (26).

## Results

### RNF20 Alters the Expression of EMT Markers and Enhances Breast Cancer Cell Migration and Invasion *In Vitro*

**Figure 1:**
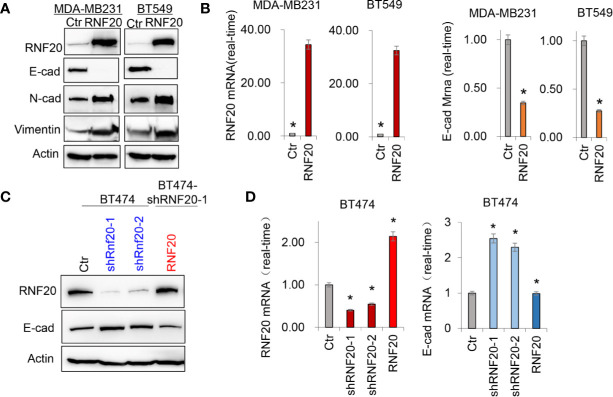
RNF20 alters the expression of epithelial-mesenchymal transition (EMT) markers. **(A)** Expression of E-cadherin, Vimentin and RNF20 in the MDA-MB231 and BT549 cells was analyzed by Western blotting. Actin was served as a loading control. **(B)** Expression of RNF20 and E-cadherin was analyzed by qPCR in MDA-MB231 and BT549 cells infected with empty vector (Ctr) or RNF20-expressing vector. *P < 0.01 by Student’s t test. **(C)** Expression of E-cadherin, and RNF20 was analyzed by Western blotting in BT474 cells with stable empty vector or knockdown of RNF20 expression as well as shRNF20-expressing BT474 cells infected with plvx-neo-RNF20. **(D)** Expression of RNF20 and E-cadherin was analyzed by qPCR in cells from **(C)**. *P < 0.01 by Student’s t test.

RNF20 has been found to be highly expressed in breast cancer cell lines, and is tightly associated with tumorigenic and metastatic capacity of tumor cells (27). To study the molecular function and mechanism of RNF20, we established stable transfectants with empty vector or RNF20 expression in MDA-MB231 and BT549 cells. We found that overexpression of RNF20 dramatically decreased the expression of epithelial marker E-cadherin and significantly upregulated the expression of mesenchymal markers N-cadherin and Vimentin (**Figure 1A**). Then we confirmed this observation by quantitative real-time PCR in the above cell lines. Consistently, RNF20 expression remarkably elevated the expression of E-cadherin mRNA (**Figure 1B**). We also generated stable clones with empty vector or knockdown of RNF20 expression in BT474 cells. Strikingly, knockdown of RNF20 expression restored E-cadherin expression in both mRNA and protein levels (**Figures 1C, D**). Collectively, these data suggest that RNF20 may induce EMT *via* the transcriptional suppression of E-cadherin expression in human breast cancer.

**Figure 2:**
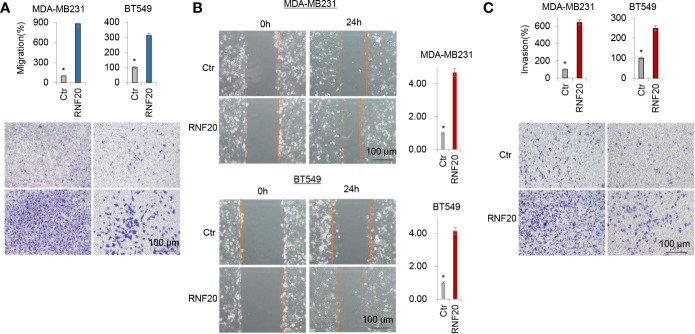
RNF20 enhances breast cancer cell migration and invasion *in vitro*. **(A–C)** Migratory **(A, B)** and invasive ability **(C)** of MDA-MB231 and BT549 cells with stable empty vector or RNF20 expression were analyzed. The percentage of migratory and invasive cells is shown in the bar graphs (mean ± SD in three separate experiments). *P < 0.01 by Student’s t test: 100 µm.

Given the tight association of RNF20 with EMT, we hypothesized that RNF20 is critical for breast cancer cells migration and invasion. As expected, RNF20 overexpression dramatically facilitated the migration and invasion of MDA-MB231 and BT549 cells in vitro (**Figures 2A–C**). Together, these observations indicate an important role for RNF20 in induction of EMT and acquisition of migratory and invasive ability in breast cancer cells.

### RNF20 Interacts With Snail

**Figure 3:**
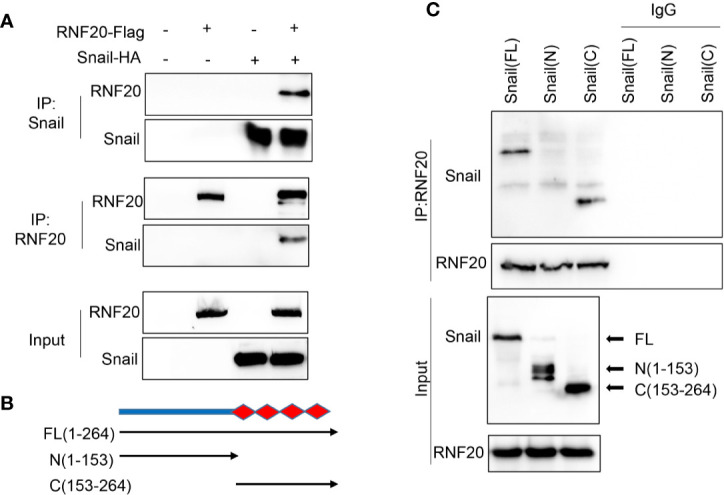
RNF20 interacts with Snail. **(A)** 293T cells were transiently coexpressed with Flag-tagged RNF20 and HA-tagged Snail. Cell extracts were immunoprecipitated separately with Flag or HA antibodies, and the associated RNF20 and Snail were examined by Western blotting, respectively. **(B)** Schematic diagram showing the structure of Snail and two deletion mutations. **(C)** Full-length and deletion mutants of Snail were coexpressed with RNF20 in 293T cells. After immunoprecipitation of RN20, associated Snail was analyzed by Western blotting.

The Snail family member Snail can trigger EMT during embryonic development and tumor progression (28–31). Because RNF20 is closely associated with suppression of E-cadherin and because Snail is a key transcriptional repressor of E-cadherin, Snail may regulate E-cadherin expression by recruiting RNF20 to E-cadherin promoter. To test this notion, we co-expressed Snail-HA and Flag-RNF20 in 293T cells and performed a co-immunoprecipitation experiment. After immunoprecipitating RNF20, we detected the associated Snail, and vice versa (**Figure 3A**). To identify the region in Snail that associates with RNF20, we generated two deletion mutants of Snail: the N-terminal region of Snail (N-Snail; amino acids 1–153) and the C-terminal region of Snail (C-Snail; amino acids 153–264) (**Figure 3B**) (32). When these two deletion mutants of Snail were coexpressed with RNF20 in 293T cells, we found that C-Snail was able to interact with RNF20, indicating that the C-terminal region of Snail is responsible for its interaction with RNF20 (**Figure 3C**).

### G9a Interacts With RNF20

**Figure 4:**
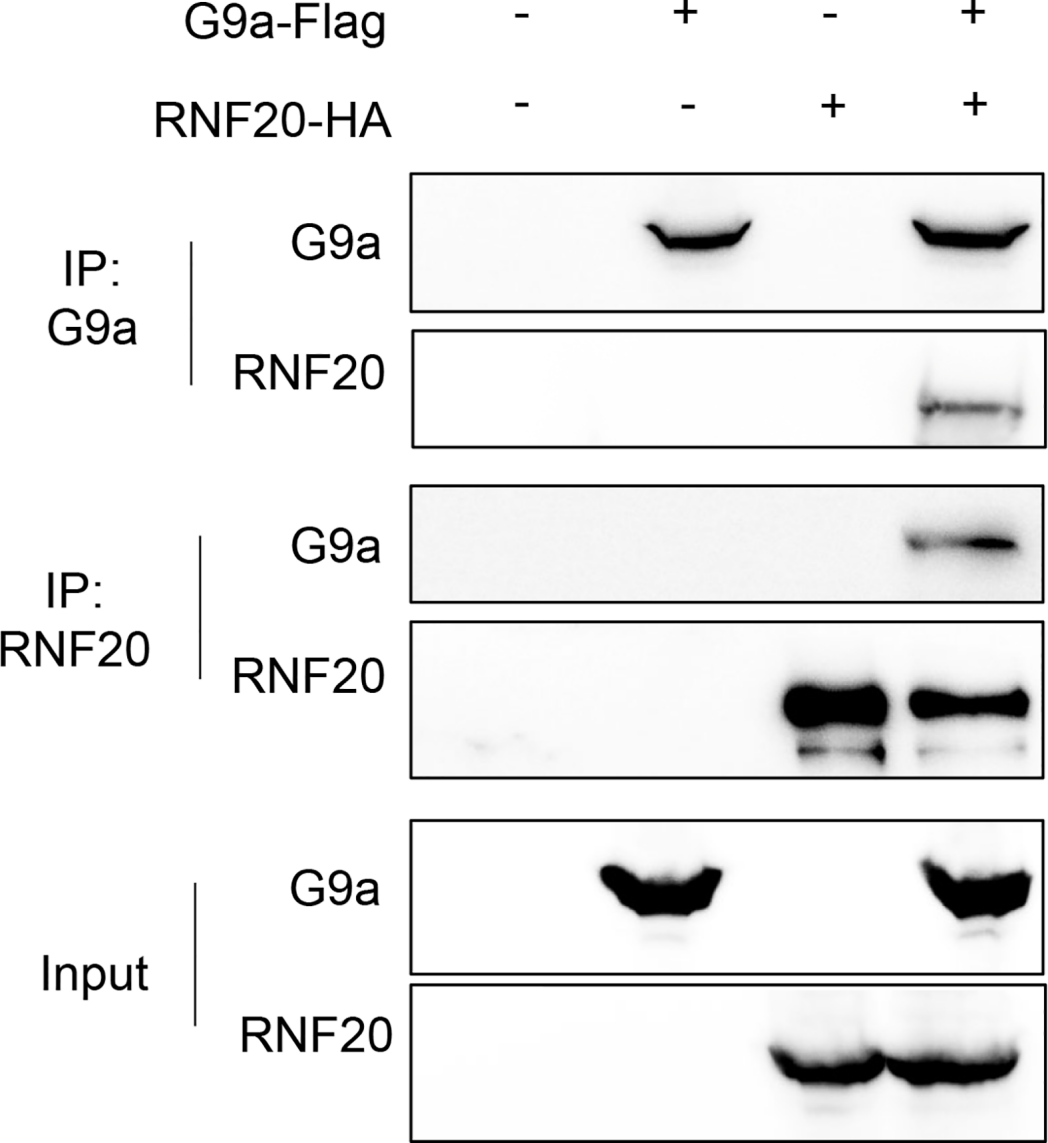
RNF20 interacts with G9a. 293T cells were transiently coexpressed with Flag-tagged G9a, HA-tagged RNF20. Cell extract were immunoprecipitated separately with Flag or HA antibodies, and the associated RNF20 and G9a were examined by Western blotting respectively.

In the previous study, we have shown that Snail recruits G9a to the E-cadherin promoter for H3K9me2 at the E-cadherin promoter in breast cancer cells (25). RNF20 is required to regulate chromosome structure by monoubiquitinating histone H2B (15, 33). Thus, RNF20 may form complex with Snail and G9a to regulate E-cadherin expression. To examine the association of RNF20 with G9a, we co-expressed RNF20-HA and G9a-Flag in 293T cells and performed a co-immunoprecipitation experiment. After immunoprecipitating RNF20, we detected the associated G9a, and vice versa (**Figure 4**). These data suggest that RNF20 may inhibit E-cadherin expression by Snail-mediated epigenetic modification.

**Figure 5:**
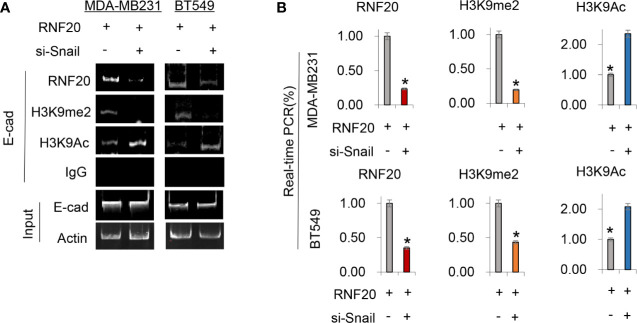
RNF20 is recruited to the E-cadherin promoter for epigenetic silencing of E-cadherin expression by Snail. **(A, B)** RNF20 was expressed in MDA-MB231 and BT549 cells, and RNF20, H3K9me2, and H3K9 acetylation at the E-cadherin promoter were analyzed by the ChIP assay. Quantitative real-time PCR was also performed to analyze ChIP samples in **(A)** (mean ± SD from three separate experiments). *P < 0.01 by Student’s t test.

To validate that the Snail-RNF20-G9a complex was associated with the E-cadherin promoter and mediated the transcriptional expression of the E-cadherin in breast cancer, we subsequently established stable clones with empty vector or RNF20 expression in MDA-MB231 and BT549. To test whether RNF20 is involved in the dynamic process of EMT induction, we investigated the association of RNF20 and the G9a-mediated H3K9me2 on the E-cadherin promoter using ChIP assays. We observed that RNF20 and H3K9me2 were enriched on the promoter of E-cadherin promoter, whereas knockdown of Snail expression significantly reduced the enrichment of RNF20 andH3K9me2, and increased level of H3K9 acetylation on the E-cadherin promoter (**Figure 5A**). Similar results were identified by the quantitative real-time PCR (**Figure 5B**). Together, these results suggest that RNF20 is recruited to the E-cadherin promoter for epigenetic silencing of E-cadherin expression in a Snail-dependent manner.

### RNF20 Promotes Tumorsphere Formation and Tumorigenicity *In Vitro*

**Figure 6:**
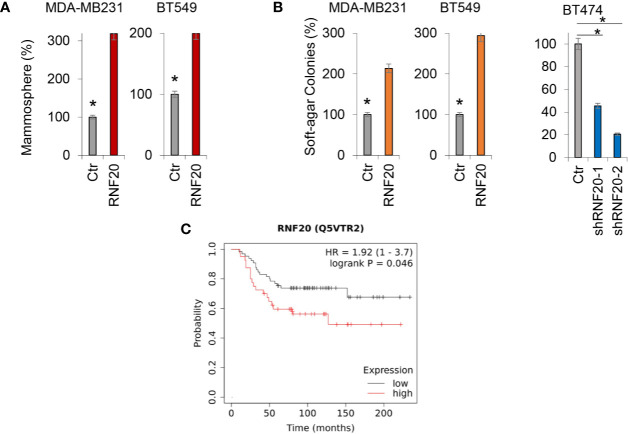
RNF20 promotes tumorsphere and colony formation. **(A)** Tumorsphere formation of MDA-MB231 and BT549 cells with stable empty vector (Ctr) or overexpression of RNF20 was measured. **(B)** The formation of colonies was measured from MDA-MB231 and BT549 cells with stable empty vector (Ctr) or overexpression of RNF20, as well as BT474 cells with stable empty vector (Ctr) or knockdown of RNF20 expression. Data are presented as a percentage of empty vector cells lines (mean ± SD in three separate experiments). *P < 0.01 by Student’s t test **(C)** Kaplan-Meier survival analysis for overall survival (OS) of patients in a breast cancer dataset according to RNF20 protein expression status. The p value was determined using the log-rank test.

Growing evidence has shown that the EMT program endows cells with stem cell-like properties, promoting tumor initiation and metastasis. As RNF20 expression contributes to the induction of EMT, we speculated that RNF20 expression might confer stem cell–like properties to breast cancer cells. To test this notion, we examined tumorsphere formation of these cells. Strikingly, RNF20 expression enhanced tumorsphere formation in MDA-MB231 and BT549 cells (**Figure 6A**). We then examined the effect of RNF20 expression on the *in vitro* tumorigenicity using soft agar assay. Indeed, RNF20 expression led to a marked increase of colony formation in MDA-MB231 and BT549 cells, whereas knockdown of RNF20 expression caused a dramatic decrease of colonies in BT474 cells (**Figure 6B**). Given the importance of RNF20 expression in breast cancer, we performed Kaplan-Meier analyses to determine the clinical relevance of RNF20 by analyzing a proteogenomic dataset that contains 65 breast tumor samples (34). Patients were divided into two groups according to RNF20 expression, with high RNF20 expression having shorter overall survival (OS) (**Figure 6C**). These data support the critical role of RNF20 in breast cancer.

## Discussion

The histone code hypothesis predicts that the post-translational modification of histones can alter the chromatin state, and therefore contributes to a key regulatory role in chromatin biology (35–37). Ubiquitination of histone H2B(H2Bub), catalyzed by the heterodimeric ubiquitin ligase complex RNF20, regulates multiple molecular and biological processes (15, 27, 38–41). H2Bub alters nucleosome stability, nucleosome reassembly and higher order compaction of the chromatin. There is growing evidence that H2Bub is a cotranscriptional event regulating histone H3 methylation at lysines 4 and 79 (10, 14, 42, 43). Recent report has shown that RNF20 can cause transcriptional suppression of some genes (21, 41, 42). Our study identified that RNF20 formed a complex with Snail and G9a. Snail-RNF20-G9a complex might regulate the expression of E-cadherin and then mediate the EMT progress. Indeed, we found that RNF20 was recruited to the promoter of E-cadherin by the interaction with Snail, which may drive histone H2B monoubiquitylation. Ubiquitination of histone H2B by RNF20 is a prerequisite for DNA methylation and gene silencing (10, 14, 42, 43). G9a also can be recruited to the E-cadherin promoter by Snail for H3K9me2 that is a marker for gene silencing (24). Together, the epigenetic regulation mediated by Snail-RNF20-G9a complex results in the suppression of E-cadherin expression.

Many solid tumors including breast cancer contain a small population of CSCs, which contribute to tumor progression. Increasing evidence has shown that EMT program confers tumor cells with CSC properties (44–46). Consistent with this notion, RNF20 expression caused the increased CSC properties by activating the EMT program in breast cancer, indicating the importance of RNF20 in controlling the viability of CSCs. CSCs are tightly associated with tumor progression (47–49). Indeed, our studies showed that RNF20 expression significantly increased tumorigenicity *in vitro*, migration and invasion of breast cancer cells. Clinically, high RNF20 expression predicted shorter survival in breast cancer patients. Clearly, our study supports that RNF20 is crucial for the aggressiveness of breast cancer cells.

Identification of the molecular targets may contribute to the development of tailored therapies to improve breast cancer patient outcomes. Given the key roles of RNF20 in EMT, CSCs, and breast cancer, RNF20 may provide potential targets for controlling breast cancer progression.

## Data Availability Statement

The datasets presented in this study can be found in online repositories. The names of the repository/repositories and accession number(s) can be found below: https://www.ncbi.nlm.nih.gov/gene/56254, Gene ID: 56254.

## Author Contributions

DW designed and performed most of experiments. YW generated the G9a plasmids. XW generated the Snail plasmids. JL and XK performed the bioinformatic analysis. CD and JL supervised the work. All authors contributed to the article and approved the submitted version.

## Funding

This work was supported by grants from Key Program of Zhejiang Province Natural Science Foundation (No. LZ17H160002 to CD), Natural Science Foundation of China (No. 81972456, 81772801 and 81472455 to CD; No. 32060163 to XW), and National Key R&D Program of China (No. 2016YFC1303200 to CD), and the Initial Research Funds for the PhD of Zunyi Medical University (No. F-932 to XW).

## Conflict of Interest

The authors declare that the research was conducted in the absence of any commercial or financial relationships that could be construed as a potential conflict of interest.
